# Optimizing IOL Calculators with Deep Learning Prediction of Total Corneal Astigmatism

**DOI:** 10.3390/jcm13185617

**Published:** 2024-09-22

**Authors:** Avi Wallerstein, Jason Fink, Chirag Shah, Damien Gatinel, Guillaume Debellemanière, Mark Cohen, Mathieu Gauvin

**Affiliations:** 1 Department of Ophthalmology and Visual Sciences, McGill University, Montreal, QC H3A 0G4, Canada; mgauvin@lasikmd.com; 2 LASIK MD, Montreal, QC H3B 4W8, Canada; mcohen@lasikmd.com; 3 The Philadelphia College of a Osteopathic Medicine, Philadelphia, PA 19131, USA; 4 LASIK Experts, Princeton, NJ 08648, USA; shahlasikmd@gmail.com; 5 Department of Ophthalmology, Rothschild Foundation Hospital, 75019 Paris, France; gatinel@gmail.com (D.G.);; 6 Department of Ophthalmology, Université de Sherbrooke, Sherbrooke, QC J1K 2R1, Canada; 7 Department of Electrical Engineering, École de Technologie Supérieure, Montreal, QC H3C 1K3, Canada

**Keywords:** deep learning, machine learning, artificial intelligence, total corneal astigmatism, intraocular lens, refractive surgery

## Abstract

**Background/Objectives**: This study aims to identify the most accurate regression model for predicting total corneal astigmatism (TCA) from anterior corneal astigmatism (ACA) and to fine-tune the best model’s architecture to further optimize predictive accuracy. **Methods**: A retrospective review of 19,468 eyes screened for refractive surgery was conducted using electronic medical records. Corneal topography data were acquired using the Pentacam HR. Various types (7) and subtypes (21) of regression learners were tested, with a deep neural network (DNN) emerging as the most suitable. The DNN was further refined, experimenting with 23 different architectures. Model performance was evaluated using root mean squared error (RMSE), R^2^, average residual error, and circular error. The final model only used age, ACA magnitude, and ACA axis to predict TCA magnitude and axis. Results were compared to predictions from one of the leading TCA prediction formulas. **Results**: Our model achieved higher performance for TCA magnitude prediction (R^2^ = 0.9740, RMSE = 0.0963 D, and average residual error = 0.0733 D) compared to the leading formula (R^2^ = 0.8590, RMSE = 0.2257 D, and average residual error = 0.1928 D). Axis prediction error also improved by an average of 8.1° (average axis prediction error = 4.74° versus 12.8°). The deep learning approach consistently demonstrated smaller errors and tighter clustering around actual values compared to the traditional formula. **Conclusion**: Deep learning techniques significantly outperformed traditional methods for TCA prediction accuracy using the Pentacam HR. This approach may lead to more precise TCA calculations and better IOL selection, potentially enhancing surgical outcomes.

## 1. Introduction

Accurate measurement or prediction of total corneal astigmatism (TCA) is crucial for selecting the appropriate power and orientation (axis) of toric intraocular lenses (IOLs) or toric implantable collamer lenses (ICLs) [1]. Emphasis has been placed on measuring anterior corneal astigmatism (ACA) from the easily accessible anterior surface for surgical planning. However, posterior corneal astigmatism (PCA) also contributes significantly to TCA, impacting optimal IOL selection.

Since PCA resides on the less accessible posterior corneal surface, current measurement devices introduce greater variability leading to lower accuracy of PCA readings [2,3,4]. Consequently, population averages and TCA prediction models have become valuable alternatives [5,6]. Classical linear regression formulas are the basis for predicting TCA from ACA. Research also suggests the benefit of using predicted TCA over measured TCA, which is thought to be due to the inherent inaccuracies associated with measuring PCA or TCA directly [7,8]. Only two studies have advocated for measured TCA over predicted TCA, while more studies support the use of predicted TCA [9,10].

Accurate IOL power prediction ensures successful surgical outcomes by minimizing over- or under-corrections, reducing residual refractive errors that cause visual distortions, and ultimately enhancing patient satisfaction. Linear-regression-based formulas like the Abulafia–Koch formula (A–K model) (developed with data from 78 eyes) exist for TCA prediction [7]. However, only one model, the Kane formula (validated with 823 eyes), incorporates machine learning, specifically using an extreme gradient boosting algorithm [11]. The Barrett Universal II (TCA prediction method is undisclosed, reported as “a black box”) and Kane formulas both use keratometry, axial length, and anterior chamber depth as core inputs [12,13]. The Kane formula additionally incorporates lens thickness and central corneal thickness as standard parameters in its calculations [13]. Neither the A–K nor Barrett Universal II and Kane formulas use patient age in their standard formulas [12,13]. Age is a factor that significantly influences the magnitude and orientation of all corneal astigmatism components [14,15]. Although subtle, there are complex age-related changes that can potentially impact the accuracy of toric IOL calculations [15].

In this study, we leverage machine learning techniques to predict more accurate TCA values by utilizing ACA measurements obtained from the Pentacam HR in nearly 20,000 eyes. Machine learning algorithms excel at training on massive datasets, enabling them to make robust predictions on unseen data. Our large and diverse dataset provided the ideal platform for this endeavor.

## 2. Patients and Methods

A retrospective, cross-sectional electronic medical record database review of consecutive patients seeking corneal and intraocular refractive procedures at a single refractive surgery center yielded 19,468 eyes from patients who were candidates for refractive surgery at a single site. In this study, all patients were included regardless of whether they underwent surgery, unless they were deemed non-candidates by trained eyecare professionals, who would then flag the EMR chart with that designation. The criteria were those being used by a group of 20 surgeons in a large multi-center practice. Naturally occurring corneal irregularities (irregular astigmatism on topography) were not exclusion criteria. If one eye had a corneal or ocular disease that disqualified the patient from refractive surgery, both eyes were excluded. Those with a history of previous ocular and/or corneal surgery noted in the EMR chart were excluded. The inclusion/exclusion was managed through automated screening filters in our large EMR database. Eyes with extreme ACA values exceeding 5 D were manually reviewed for diseased corneas or artifactual Pentacam HR measurements and excluded as necessary. The study adhered to the tenets of the Declaration of Helsinki and was approved by the Ethics Review Board of the Canadian Ophthalmic Research Centre (CORC-RS2020-005) in February 2020. All patients provided consent for the use of anonymized data for research.

### 2.1. Data Acquisition

Preoperative corneal topography data were acquired in all patients using the Pentacam HR (OCULUS, Wetzlar, Germany). The dataset included age, gender, central corneal thickness, K1 (min keratometry) and K2 (max keratometry) at a 3 mm diameter, eye (OD/OS), index of surface variance (ISV), index of vertical asymmetry (IVA), keratoconus index (KI), center keratoconus index (CKI), index of height asymmetry (IHA), ACA magnitude, ACA axis, TCA magnitude, and TCA axis.

### 2.2. Model Development and Tested Regression Learners

Our international research team has vast experience with machine learning, prediction, and optimization applied to refractive surgery data [16,17,18,19,20,21]. Our analysis process began with data collection from the Pentacam HR, followed by the selection of variables for testing. Initial model testing was conducted using MATLAB R2024a software (The MathWorks). Various regression models were trained and evaluated to determine the best-performing model for the given data. These included linear regression models (linear regression, stepwise linear regression, and robust linear regression), tree-based models (regression trees and ensemble of regression trees including boosting, bagging, and random forest), support vector machines (SVM with linear, quadratic, cubic, and Gaussian kernels), Gaussian process regression (GPR with rational quadratic, squared exponential, Matern 5/2, and exponential kernels), neural networks (shallow and deep), generalized additive models (GAM), and ensemble methods (bagged trees, boosted trees, and random forest). The deep neural network (DNN) model emerged as the most suitable. Further refinement of the predictions was achieved by experimenting with 23 different DNN architectures using the PyTorch library in Python. To identify the most accurate regression models and optimize architecture tuning to predict TCA from ACA measurements, we prioritized a model that maximized R^2^ and Test Accuracy while minimizing Test Loss and RMSE. To do so, a global metric of performance was calculated as *R^2^ + Test Accuracy − Test Loss − RMSE.* Our experiments included both deep and shallow neural network architectures. Although initial testing in MATLAB indicated that deep neural networks performed best, our subsequent experiments with both shallow and deep neural network architectures in PyTorch revealed that increasing the depth of the network did not significantly improve the model’s prediction performance for TCA prediction. During the model development process, it was found that corneal thickness, gender, eye (OD/OS), K1, K2, ISV, IVA, KI, CKI, and IHA did not significantly improve prediction accuracy and only added complexity to the model. Therefore, these variables were excluded from the final model to maintain simplicity and ease of use for surgeons.

### 2.3. Data and Statistical Analysis

Regression analysis was employed to evaluate the model’s performance, with metrics such as root mean squared error (RMSE), R^2^, and average residual error used to assess the accuracy of the model’s TCA magnitude predictions. Circular error, which quantifies the average angular deviation between the predicted and actual TCA axis values, was used to evaluate the accuracy of the TCA axis predictions. This study utilized three distinct approaches to predict TCA magnitude and axis. The first method involved direct training using the Pentacam HR data. The second and third methods both began by splitting the Pentacam HR data into components but differed in their approach. The second method predicted TCA magnitude using Cartesian components x = M cos(θ) and y = M sin(θ), while the third method employed power vector components J0 = M cos(2θ) and J45 = M sin(2θ). In both latter methods, the TCA magnitude was subsequently reconstructed from the predicted components. This multi-faceted approach allowed for a comprehensive evaluation of different predictive techniques for TCA. For each approach, the models’ predictions were compared to the A–K formula. This is a leading regression-based method used to estimate TCA from anterior corneal measurements. It was developed to account for the contribution of PCA in toric intraocular lens calculations. The A–K formula employs separate linear regression equations for the x and y vector components of corneal astigmatism. For the x-component, the regression is x’-component = 0.508 + 0.926x-compoent, while, for the y-component, it is y’-component = 0.009 + 0.932y-component [7]. These equations are applied to the measured ACA components to estimate TCA.

## 3. Results

### 3.1. Corneal Astigmatism Components

The mean magnitude and axis mode of corneal astigmatism were 1.06 ± 0.74 D at 90.04° for ACA (range: 0–6.6 D; axis 0 to 180°), 0.33 ± 0.16 D at 88.82° for PCA (range: 0–1.2 D; axis 0 to 180°), and 0.81 ± 0.60 D at 89.64° for TCA (range 0 to 6.5 D; axis 0 to 180°). ACA was with-the-rule (WTR) in 77.6%, against-the-rule (ATR) in 10.6%, and oblique in 11.84% of eyes. PCA was WTR in 1.74%, ATR in 94.2%, and oblique in 4.06% of eyes. TCA was WTR in 67.4%, ATR in 16.81%, and oblique in 15.8% of eyes. Most eyes (79.2%) exhibited WTR ACA and TCA with ATR PCA. The mean age of participants was 36.32 ± 12.82 years (range: 18–87 years).

### 3.2. Determining the Best Predictors and Machine Learning Architectures for TCA Prediction

ACA magnitude, ACA axis, and age were used as predictors for TCA magnitude and TCA axis, using a distinct model for magnitude and axis. The inclusion of additional variables such as gender, corneal thickness, K1 (3 mm), K2 (3 mm), eye (OD/OS), index of surface variance (ISV), index of vertical asymmetry (IVA), keratoconus index (KI), center keratoconus index (CKI), and index of height asymmetry (IHA) did not improve the prediction accuracy of our machine learning models and were therefore discarded. We investigated the performance of various machine learning methods, including random forests, bagging, principal component analysis, polynomial regressions, artificial neural networks, and deep learning. Initial results confirmed that deep learning outperformed the other methods. Subsequently, we explored 23 different neural network architectures (Table 1), varying in network size (number of layers), activation function type, hidden layer dimension (number of neurons), number of batch normalization layers, and inclusion of L2 regularization with weight decay. Of our dataset, 80% (15,574 eyes) was used for training and 20% for testing (3,894 eyes). Consistently accurate predictions of TCA were achieved across all architectures (Table 1).

From the evaluated neural network architectures, we prioritized a model that maximized R^2^ and Test Accuracy while minimizing Test Loss and RMSE using a global metric of performance. Our global metric was the highest for model #1, making it the optimal choice for our TCA prediction application (see Table 1 for details on models’ architecture).

### 3.3. Comparison of Conventional A–K Formula to Our Deep Learning Model

**Figure 1:**
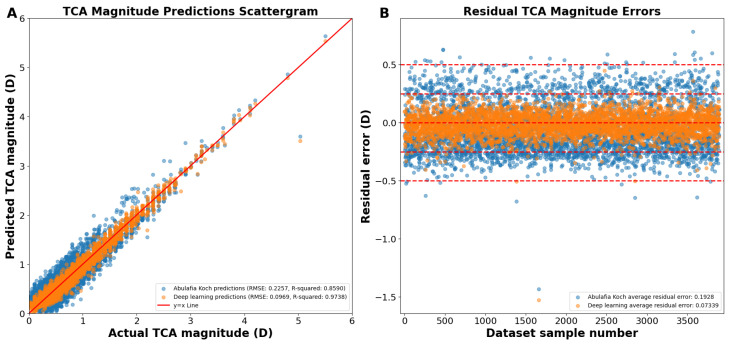
(**A**) Actual vs. predicted total corneal astigmatism magnitude using deep learning without J0/J45 astigmatism decomposition (orange dots) and the A–K formula (blue dots). (**B**) Residual error plots for the A–K formula and the deep learning model. Residual error plots visually assess the quality of the fitted regression model, depicting how closely the model’s predicted values align with the actual data points. The residual error for each data point is calculated as the difference between its predicted value and the corresponding actual value. Abbreviations: TCA = total corneal astigmatism, RMSE = root mean square error.

As shown on the actual versus predicted TCA magnitude scattergram, the A–K formula achieved a Pentacam HR TCA prediction with a root mean square error (RMSE) of 0.2257 D and an R^2^ of 0.8590 (Figure 1A,B). Our deep learning approach had an RMSE of 0.0969 D and an R^2^ of 0.9738 (Figure 1A,B). The average absolute residual error was of 0.1928 ± 0.1173 D for A–K formula and 0.0734 ± 0.0958 D for the deep learning model (*p* < 0.0001; Figure 1B). Residual error plots showed that 68.80% of the A–K formula’s predictions and 98.84% of the deep learning model’s predictions were within an error of 0.25 D (*p* < 0.0001; Figure 1B), with 99.08% and 99.92% within an error of 0.50 D (*p* < 0.0001), respectively.

**Figure 2:**
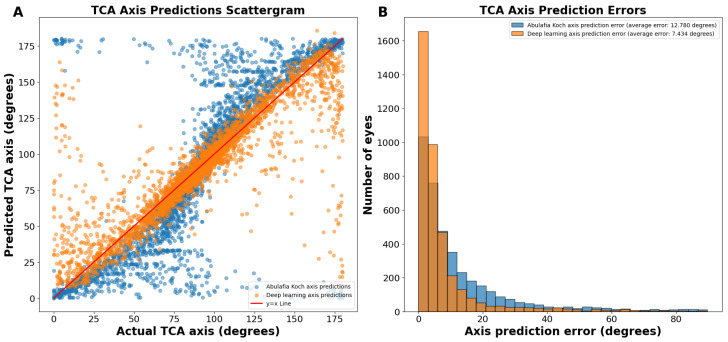
(**A**) Actual vs. predicted total corneal astigmatism axis using deep learning without J0/J45 astigmatism decomposition (orange dots) and the A–K formula (blue dots). (**B**) Total corneal astigmatism axis prediction errors using deep learning without J0/J45 astigmatism decomposition and the A–K formula. Abbreviations: TCA = total corneal astigmatism.

On the actual versus predicted TCA axis scattergram, the A–K predictions were sigmoidal in nature, showing underprediction (predictions below the y = x line) between 0 and 89 degrees and overprediction (predictions above the y = x line) between 91 and 180 degrees (Figure 2A). In contrast, deep learning predictions were more linear (Figure 2A).

The average absolute circular axis error was 12.78 ± 16.04 degrees for the A–K formula and 7.43 ± 11.75 degrees for the deep learning model (*p* < 0.0001; Figure 2B). The distribution of error bins, each representing 3 degrees, indicated that the A–K formula resulted in a higher frequency of large TCA axis prediction errors, whereas the deep learning model predominantly produced small errors. Specifically, there were 60.1% more eyes with an error of 0 to 3 degrees using the deep learning model compared to the A–K formula.

### 3.4. The Best Model: Deep Learning with J0 and J45 Astigmatism Component Split

**Figure 3:**
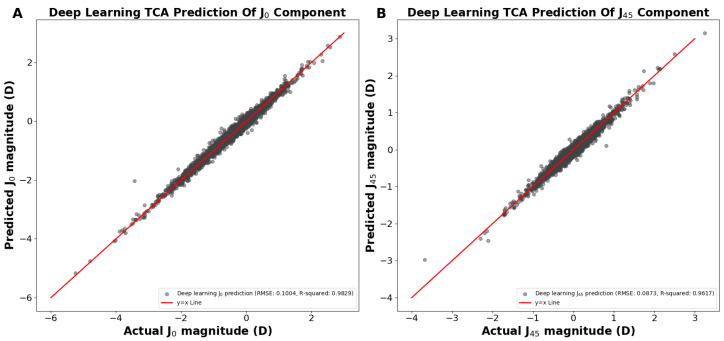
(**A**) Actual vs. predicted total corneal astigmatism magnitude using deep learning for the J0 component. (**B**) Actual vs. predicted total corneal astigmatism magnitude using deep learning for the J45 component. Abbreviations: TCA = total corneal astigmatism, RMSE = root mean square error.

In the above demonstrations (Figure 1 and Figure 2), we achieved TCA predictions on ACA astigmatism data without first splitting ACA into its primary J0 and J45 components. Utilizing the J0 and J45 method for splitting the ACA before training significantly improved our TCA predictions. We developed two separate models to predict the TCA J0 and TCA J45 components, using age and their corresponding ACA J0 and ACA J45 components as predictors, respectively. Using the predicted J0 and J45 TCA predictions, the Pythagorean trigonometric formula and arctangent formula were used to reconstruct the TCA magnitude and TCA axis, respectively. Our deep learning models predicted the J0 and J45 TCA components, resulting in a predicted J0 TCA component prediction RMSE of 0.1004 D and R^2^ of 0.9829 (Figure 3A) and a J45 component prediction RMSE of 0.0895 D and R^2^ of 0.9597 (Figure 3B).

**Figure 4:**
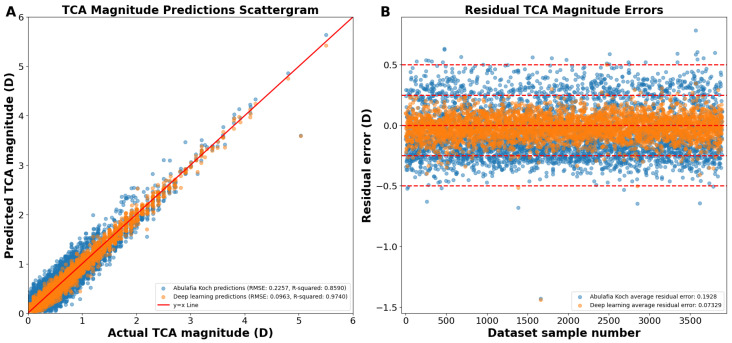
(**A**) Actual vs. predicted total corneal astigmatism magnitude using deep learning with J0/J45 astigmatism reconstruction (orange dots) and the A–K formula (blue dots). (**B**) Residual prediction errors using deep learning with J0/J45 astigmatism reconstruction and the A–K formula. Abbreviations: TCA = total corneal astigmatism, RMSE = root mean square error.

Reconstructing the TCA from the J0 and J45 TCA components yielded an overall RMSE of 0.0963 D, R^2^ of 0.9740, and an average residual error of 0.0733 D (Figure 4A). Residual error plots showed that the deep learning prediction errors had 98.7% of the prediction errors within 0.25 D compared to 68.8% for A–K formula (*p* < 0.0001; Figure 4B). For deep learning prediction errors, 99.9% were within 0.50 D versus 99.1% for the A–K formula (*p* < 0.0001). The average absolute residual error for the reconstructed deep learning J0/J45 components was 0.0733 ± 0.0625 D versus 0.1928 ± 0.1173 D for the A–K formula (*p* < 0.0001; Figure 4B).

**Figure 5:**
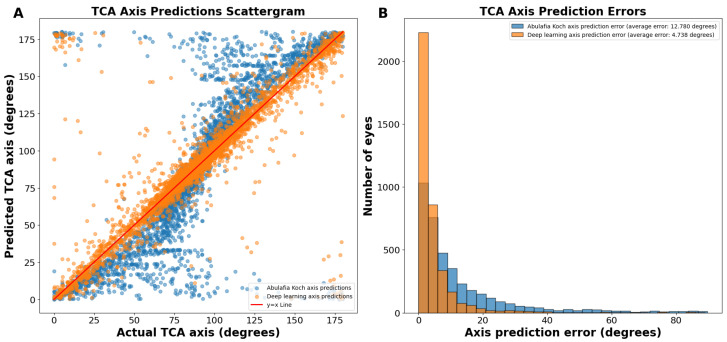
(**A**) Actual vs. predicted total corneal astigmatism axis using deep learning with J0/J45 astigmatism decomposition (orange dots) and the A–K formula (blue dots). (**B**) Total corneal astigmatism axis prediction errors using deep learning with J0/J45 astigmatism decomposition and the A–K formula. Abbreviations: TCA = total corneal astigmatism.

The J0/J45 split method yielded axis predictions with an even more linear trend than our deep learning predictions without splitting the astigmatism into J0 and J45 components (Figure 5A). The average absolute circular axis error was 12.78 ± 16.04 degrees for the A–K formula and 4.738 ± 8.069 degrees for the J0/J45 split deep learning model (*p* < 0.0001; Figure 5B). Specifically, there were 115.5% more eyes with an error of 0 to 3 degrees using this deep learning model compared to the A–K formula. This method had a 36.3% reduction in average prediction circular error from the standard deep learning axis prediction method.

Deep learning with J0 and J45 astigmatism component split was therefore the best methodology and yielded predictions that were vastly superior to the A–K formula when applied to the same test dataset of 3894 eyes. Table 2 provides a summary of our final results for TCA magnitude and axis predictions, comparing the performance of the Abulafia–Koch formula, our deep learning model without J0 and J45 component splitting, and our optimized deep learning model with J0 and J45 component splitting, demonstrating progressive improvements in prediction accuracy across all metrics.

## 4. Discussion

### 4.1. Importance of Accurate TCA Prediction

Accurate TCA prediction is critical for successful IOL selection and optimal visual outcomes post-surgery. TCA encompasses both ACA from the accessible anterior corneal surface and PCA from the less accessible posterior surface. Toric IOL calculators rely on TCA values to determine the ideal power and orientation of the lens for correcting astigmatism. Highly accurate TCA predictions minimize residual refractive errors, enhancing patient satisfaction and surgical success. Measuring PCA through the corneal stroma may introduce “measurement noise”, resulting in less accurate TCA values for IOL selection. It is worth noting that the lower accuracy in PCA measurements from devices is exacerbated in low-income areas where access to advanced topographers is limited. This further emphasizes the importance of developing accurate prediction models that can work with more widely available ACA data. The current deep learning approach for predicting TCA offers several significant differences compared to the A–K formula: it utilizes Pentacam HR data and a much larger and more diverse dataset of 19,468 eyes (versus 78 eyes), allowing for more robust and generalizable Pentacam HR TCA predictions. The deep learning model also uniquely incorporates age as a potential predictor. The combination of deep learning, the incorporation of age, and using J0 and J45 astigmatism component splitting enhanced prediction accuracy, with lower RMSE and average absolute residual error and higher R^2^ values for both TCA magnitude and axis predictions. The axis prediction was also significantly improved when evaluating the average absolute circular error of both models.

### 4.2. Difference between an IOL Calculator and a TCA Prediction Formula

IOL calculators like Barrett Universal II, Kane, Holladay, Haigis, EVO, Hoffer QST, SRK/T, and Hill-RBF are comprehensive tools designed to predict the optimal intraocular lens power for cataract surgery, aiming to achieve the desired refractive outcome. These calculators use various biometric measurements and formulas to estimate the effective lens position and required IOL power. While some advanced calculators like Barrett Universal II have built-in methods to account for TCA, others may not fully address PCA. On the other hand, TCA predictors like the A–K model specifically focus on estimating the TCA by accounting for both anterior and posterior corneal surfaces. These TCA predictors can be used to enhance the accuracy of IOL calculators that do not inherently account for PCA, thereby improving the prediction of residual corneal astigmatism after IOL implantation. By incorporating TCA predictors, surgeons can potentially achieve more precise astigmatism correction during cataract surgery, especially when using toric IOLs. The incorporation of the A–K formula, significantly improved residual corneal astigmatism prediction for Alcon and Holladay toric IOL calculators, bringing their performance in line with the Barrett Universal II formula which has its own built-in TCA prediction. PhysIOL has integrated this advancement by offering the option to include the A–K formula in their toric IOL calculator. In our study, we chose the A–K model as a comparison benchmark because it is one of the leading publicly disclosed formulas for TCA prediction. It is worth noting that, while many IOL calculators use TCA prediction, they typically do not disclose their specific TCA prediction algorithms or formulas publicly.

### 4.3. Challenges and Limitations with Existing Solutions

Two popular approaches for estimating TCA are the Barrett Universal II and the A–K formulas. The A–K formula relies on a published mathematical model that includes regression analysis. In contrast, the Barrett Universal II uses an unpublished “black box” algorithm, the details and quantified accuracy of which are not publicly known nor have they been published. Neither the A–K formula nor the Barrett Universal II formula incorporates age as a variable in their TCA predictions. This is evident from the known details of the A–K formula and confirmed by the absence of an age input in the Barrett Universal II calculator. The Kane formula uses AI but its detailed methodology remains unpublished, including whether age was used in training. Smaller or less diverse datasets can be vulnerable to prediction errors, especially when machine learning methods are employed. However, Darcy et al. mentions that the Kane formula was developed using approximately 30,000 cases from selected refractive cataract practices, but data or details about these cases were never disclosed nor peer-reviewed [13]. Our deep learning model, trained on a diverse dataset of 19,468 eyes, currently represents the largest datasets and peer-reviewed paper for TCA magnitude and axis prediction.

Age is a potential factor often overlooked in existing solutions regarding its influence on astigmatism. Studies indicate a trend towards elevated TCA magnitude with ATR astigmatism axis as age increases [6,14,22,23,24]. A more recent study also reported that TCA starts high from age 18 to 23, then decreases steadily until age 54 to 59, before rising quickly by 32% in only the next decade from 60 to 70+ years old [15]. Our deep learning model incorporates a wide age range during training, enhancing prediction accuracy. While the inclusion of age only marginally improved the accuracy of our model, there are compelling reasons to retain it as a variable. Firstly, the residual plots show a qualitative improvement with age included, demonstrating a tighter clustering around the ideal prediction line and a slight reduction in prediction errors for higher astigmatism values. Secondly, age has well-established clinical relevance in corneal astigmatism, as evidenced by our previous research showing age-dependent relationships between ACA and PCA. More specifically, we have shown that, in WTR ACA eyes, the correlation between ACA and PCA decreases from R = 0.78 at 18 yo to R ≤ 0.48 in eyes ≥ 65 yo. Oblique ACA eyes showed lower correlations, peaking at R = 0.51 at 24 yo and decreasing to R = 0.02 after 72 yo. ATR ACA eyes showed a mild positive correlation in midlife (R = 0.15; 41 yo), switching to a moderate inverse correlation in older age (R = −0.3461; ≥ 72 yo) [15].

As an ad hoc demonstration, we investigated the relationships between ACA, TCA, and age (Supplementary Figures S1 and S2). Both figures present three plots representing against-the-rule (ATR), with-the-rule (WTR), and oblique astigmatism, with ACA on the x-axis and age on the y-axis. The color scale represents the difference between TCA and ACA (TCA–ACA). Supplementary Figure S1 uses a simplified red and blue color scheme so that the general trends in the relationship between ACA, TCA, and age may be observed. For ATR astigmatism, most data points appear red, indicating that TCA is generally larger than ACA, except at very high ACA values. In contrast, the WTR plot shows bluer overall, suggesting that TCA is often smaller than ACA for WTR astigmatism. However, Supplementary Figure S2 uses a more complex color scale, showing more nuanced details in the magnitude of the difference between ACA and TCA. This visualization reveals that, while TCA may be larger than ACA in many cases, especially for ATR astigmatism, the difference is often relatively small. The more detailed color scale shows that TCA, when larger than ACA, is rarely much higher than ACA. The complex color scale allows for the appreciation of subtle variations in the TCA–ACA relationship across astigmatism magnitudes and orientations. For instance, in the ATR plot, we can see a gradual transition from blue (positive TCA–ACA) to green and yellow (smaller negative TCA–ACA) as ACA increases, rather than a stark contrast between the red and blue plot. The A–K model, which uses only 78 eyes, undeniably has large 95% confidence intervals for the slope and intercept, impacting its reliability. In addition, its dataset likely only consisted of older eyes, as these were all patients undergoing cataract extraction. The A–K model was also not specifically trained to predict Pentacam HR data, as in the current paper, which further explains the superior accuracy here. The A–K formula, however, remains an excellent time-tested approach and could potentially improve its predictions by considering age and, more importantly, by applying machine learning techniques on a larger dataset.

To our knowledge, only one previous study used a very large corneal dataset, using the IOLMaster 700 with total keratometry module, to predict total J0 and J45 corneal astigmatism from anterior corneal data [5]. In comparison to this earlier work, our study utilized the Pentacam HR, which has been shown to yield the highest repeatability in corneal astigmatism measurements, with highly repeatable total corneal measurements compared to five devices, including the former IOLMaster 500, which, at the time of the study, had the lowest repeatability among the five devices [25]. The calculation principle of corneal curvature of the IOLMaster 700 is similar to the IOLMaster 500. The IOLMaster 700 projects 18 spots on three areas of the cornea and those areas are distributed within 6 spots [25]. In contrast, the Pentacam HR used 125,000 corneal datapoints, a major upgrade from the former regular Pentacam, which only used 25,000 datapoints.

In contrast to this prior work, our study included a diverse array of seven machine learning methods and 21 subcategories to identify the most effective model, including linear regression models, tree-based models, support vector machines, Gaussian process regression, generalized additive models, and ensemble methods. Through this comprehensive evaluation, we found that the deep neural network consistently outperformed other methods. Subsequently, we then further optimized our model by testing 23 different deep learning architectures to achieve the best possible performance. To our knowledge, this is the first time that such a methodological approach using advanced automatic machine learning using multiple learners and sub-architectures has been applied in ophthalmology. The previous study also used data from multiple centers, potentially increasing variability.

Our approach achieved the same mean and standard deviation predictions as the previous method. However, using Pentacam HR data (considered more accurate), our prediction error, though similar, likely leads to higher overall accuracy due to the improved precision of the TCA data used for training. Furthermore, our model accomplished this same level of accuracy with only three variables (age, ACA magnitude, and axis), whereas the IOLMaster-700-based model used 10 variables (K, AL, CCT, ACD, LT, W2W, R1, R2, TR1, and TR2). Additionally, they did not report axis prediction or axis prediction error, making it difficult to fully assess their model’s performance in this critical aspect. The previous study also did not include actual vs. predicted scattergrams, further limiting the assessment of the model’s accuracy. The previous study only predicted J0 and J45, making it less practical for surgeons unfamiliar with these vectors to easily determine the required toricity.

Our inclusion of clinically relevant metrics, such as the percentage of predictions within 0.25 D and 0.50 D and visual validation with scattergrams and R^2^ values, provides a more comprehensive evaluation of our model’s performance. While both studies achieved similar mean prediction errors, our method demonstrates equivalent accuracy with significantly less complexity and a direct comparison to the well-known A–K formula, underscoring the robustness and practicality of our deep-learning-based TCA prediction method.

### 4.4. Deep Learning as a Solution

The extensive and diverse large dataset that we used offered a unique opportunity to harness deep learning for accurate TCA prediction. Deep learning algorithms excel at identifying complex patterns within large datasets, enabling the model to learn variable relationships and predict TCA with superior accuracy on unseen data. The accuracy that we achieved was unprecedented. This underscores the importance of large and diverse datasets in training robust deep learning models.

### 4.5. Astigmatism Data Splitting versus No Splitting

We initially trained the neural network on raw data (i.e., without using J0 and J45 components) and compared its performance to the A–K formula. The model already showed improved results. To further refine predictions, we explored data preprocessing techniques, testing the X, Y Cartesian split and the J0/J45 methods for splitting astigmatism into components. While the X, Y Cartesian split method did not improve performance, the J0/J45 method achieved comparable TCA magnitude predictions and magnitude error to the raw dataset model but very significantly improved TCA axis prediction accuracy.

### 4.6. Relationships between Corneal Variables

Regression plots of our TCA magnitude predictions revealed a tighter distribution, indicating significantly lower error compared to existing methods. The A–K formula displayed a sigmoidal curve for axis prediction, suggesting a tendency to under- or overestimate the astigmatism axis. While our non-preprocessed predictions (without J0/J45 split) achieved greater linearity, they still exhibited slight under- and overestimation. The refined model using J0 and J45 split yielded the most linear TCA axis predictions, further minimizing potential errors. Furthermore, in the above-reported axis prediction scattergrams (Figure 2A), points that appear as massive outliers are not outliers, as 180 degrees and 0 degrees represent the same axis for astigmatism. Ad hoc analyses revealed that the vast majority of axis predictions deviating from the ideal y = x line corresponded to cases where the ACA was less than 0.50 D (Supplementary Figure S3). While axis prediction was slightly less reliable for cases where ACA magnitude was less than 0.50 D, predictions with deep learning remained more accurate than predictions using the A–K formula. It is important to note that, while some surgeons opt for low-powered toric IOLs in patients with mild astigmatism, many choose to manage these cases by converting to a spherical equivalent correction. These findings highlight that the bulk of our axis prediction errors were associated with low astigmatism cases, which are often managed differently depending on the surgeon’s preference. To better assess the accuracy of TCA axis prediction, we therefore used a circular TCA axis error histogram (Figure 2B). These specialized histograms are ideal for visualizing the distribution of errors in predicted astigmatism axis.

### 4.7. mEYEstro TCA Predictor Software

Based on the prediction reported in the current paper we developed a user-friendly web application that integrates the current deep-learning-based calculator, providing surgeons with more accurate TCA and PCA values to facilitate optimal IOL toricity planning for their patients. Surgeons can try it free at www.refractivesurgery.ca/software.

## 5. Limitations and Future Directions

To further enhance the model’s performance, we plan to investigate the inclusion of separate datasets for WTR, ATR, and oblique astigmatism, potentially refining the model’s ability to handle the specific characteristics of each astigmatism type. While numerous variables were evaluated and subsequently excluded due to their lack of significant impact, further investigation could incorporate additional measurements such as corneal diameter, white-to-white distance, and pachymetric progression index. These parameters may offer valuable insights to complement our current findings. However, it is important to note that these variables were not available in our existing dataset, presenting an opportunity for future studies to expand upon this research. It is necessary to acknowledge that measuring PCA magnitude and axis is less reliable than ACA, particularly for smaller PCA magnitudes. Ideally, repeated measurements would enhance reliability. However, the large sample size required for a machine learning study makes this approach impractical for future research, thus presenting a limitation. Fortunately, our study’s substantial sample size helps mitigate this issue by effectively averaging out measurement noise in cases with low ACA, thereby enhancing the overall reliability of our findings. Future work may explore the adaptability of our deep learning model to other biometry devices that measure total keratometry, such as the IOL Master 700, Eyestar 900, Galilei G6, CSO Sirius+, CSO MS-39, Tomey TMS-5, and Anterion. This expansion would require appropriate training, recalibration, and testing for each device. Additionally, we are developing theoretical back-calculations to assess whether our TCA prediction could enhance IOL calculation predictions of postoperative cylinder. To validate the model’s clinical applicability across different devices, we plan to conduct studies evaluating actual post-refractive outcomes. These efforts aim to broaden the model’s utility and improve its accuracy in diverse clinical settings.

## 6. Conclusions

This study demonstrates the impact of deep learning on a large dataset to accurately predict TCA from ACA and age. Using the Pentacam HR, our deep learning model predicted TCA with greater accuracy compared to existing formulas. This approach has the potential to improve current IOL calculators and thereby minimize residual refractive errors and improve post-surgical visual outcomes, ultimately leading to greater patient and surgeon satisfaction.

## Figures and Tables

**Table 1:** Experimentation with different neural network architectures.

Model Number	Model Details	R^2^	Test Accuracy	Test Loss	RMSE (D)	Global Metric
1	4fc, 3 ReLU, hd 16, 2 bn, wd	0.9671	0.9687	0.0115	0.10722	1.81708
12	3fc, 2 ReLU, hd 32, 1 bn, wd	0.9672	0.9680	0.0114	0.1069	1.81690
5	4fc, 3 ReLU, hd 32, 2 bn	0.9668	0.9686	0.0116	0.10763	1.81617
3	4fc, 3 ReLU, hd 32, 2 bn, wd	0.9662	0.9687	0.0118	0.10867	1.81443
16	2fc, 1 ReLU, hd 16, 1 bn	0.9665	0.9675	0.0117	0.10811	1.81419
10	3fc, 2 ReLU, hd 32, 2 bn	0.9659	0.9676	0.0119	0.10903	1.81257
22	4fc, 3 ELU, hd 32, 2 bn, wd	0.9668	0.9648	0.0116	0.10761	1.81239
11	3fc, 2 ReLU, hd 16, 2 bn, wd	0.9658	0.9674	0.0119	0.1092	1.81210
13	3fc, 2 ReLU, hd 32, 2 bn, wd	0.9660	0.9645	0.0119	0.1090	1.80960
18	2fc, 1 ReLU, hd 32, 1 bn, wd	0.9648	0.9642	0.0125	0.11185	1.80465
17	2fc, 1 ReLU, hd 16, 1 bn, wd	0.9632	0.9648	0.0128	0.11334	1.80186
23	4fc, 3 Tanh, hd 16, 2 bn, wd	0.9666	0.9509	0.0116	0.10789	1.79801
2	4fc, 3 ReLU, hd 16, wd	0.9586	0.9586	0.0144	0.12019	1.78261
4	4fc, 3 ReLU, hd 32, wd	0.9586	0.9585	0.0145	0.12026	1.78234
7	3fc, 2 ReLU, hd 32	0.9570	0.9570	0.0150	0.12246	1.77654
6	4fc, 3 ReLU, hd 32	0.9567	0.9566	0.0151	0.12298	1.77522
20	4fc, 3 Sigmoid, hd 32, 2 bn, wd	0.9572	0.9531	0.0149	0.1222	1.77320
15	2fc, 1 ReLU, hd 16, wd	0.9556	0.9556	0.0155	0.12441	1.77129
9	3fc, 2 ReLU, hd 32, wd	0.9537	0.9537	0.0161	0.12704	1.76426
21	4fc, 3 LeakyReLU, hd 32, 2 bn, wd	0.9660	0.9124	0.0119	0.10899	1.75751
19	2fc, 1 ReLU, hd 32	0.9518	0.9517	0.0168	0.12974	1.75696
8	3fc, 2 ReLU, hd 16, wd	0.9517	0.9517	0.0169	0.12981	1.75669
14	2fc, 1 ReLU, hd 16	0.9458	0.9458	0.0189	0.13749	1.73521

Note Table 1: The model details column is structured with the number of fully connected layers (fc), the type of activation function used and how many layers used, the number of neurons in the hidden dimension (hd), the number of batch normalization layers used (bn), and if weight decay (wd) was used.

**Table 2:** Performance comparison of TCA prediction models: magnitude and axis metrics.

Magnitude Metrics				Axis Metrics	
Model	RMSE (D)	R^2^	Average Error (D)	Average Circular Error (Degrees)	Axis Standard Deviation (Degrees)
Abulafia-Koch	0.2257	0.8590	0.1928	12.78	16.03
Deep Learning without J0/J45 split	0.0969	0.9738	0.0734	7.43	11.75
Deep Learning with J0/J45 split	0.0963	0.9740	0.0733	4.74	8.07

## Data Availability

The original contributions presented in the study are included in the article/supplementary material, further inquiries can be directed to the corresponding author.

## Supplementary Materials

The following supporting information can be downloaded at: https://www.mdpi.com/article/10.3390/jcm13185617/s1: Figure S1: Relationship between ACA, age and “TCA minus ACA”. The plots show ATR, WTR, and oblique astigmatism. The x-axis represents ACA in diopters, the y-axis shows age, and the color scale indicates the difference between TCA and ACA (TCA minus ACA), with red representing positive values and blue representing negative values. Figure S2: Detailed Analysis of the relationship between ACA, age and “TCA minus ACA”. The plots display ATR, WTR, and oblique astigmatism. The x-axis shows ACA in diopters, the y-axis represents age, and the color scale indicates the magnitude of the difference between TCA and ACA (TCA-ACA), with a more nuanced color gradient to highlight subtle variations. Figure S3. Impact of low ACA on TCA axis prediction. Gray dots represent all data, including cases with ACA less than 0.5D. Blue dots show only cases where ACA is 0.5D or greater.
