# Supplementary figures and images for: Optimizing IOL Calculators with Deep Learning Prediction of Total Corneal Astigmatism
Source: J Clin Med. 2024 Sep 22;13(18):5617. doi: 10.3390/jcm13185617 (PMC11432570; doi:10.3390/jcm13185617)

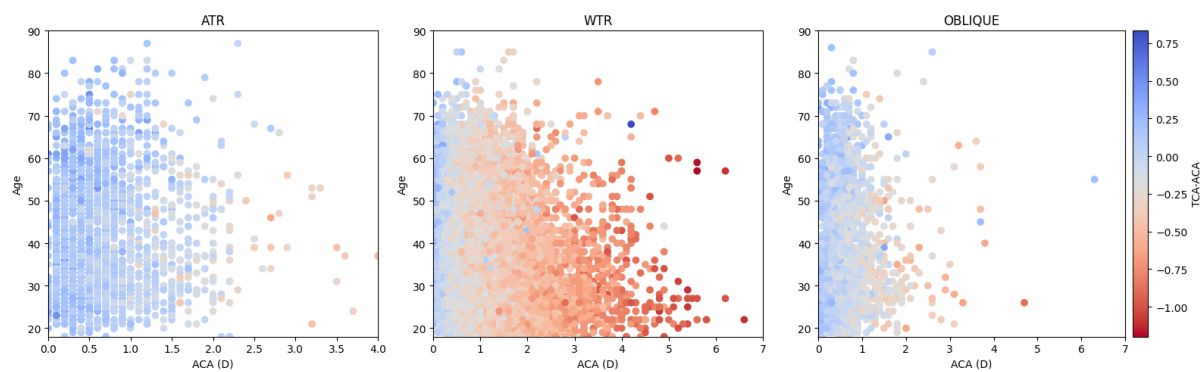

Supplement: Supplementary file 1 [file jcm-13-05617-s001.zip › Supplemental Figure S1.pdf]

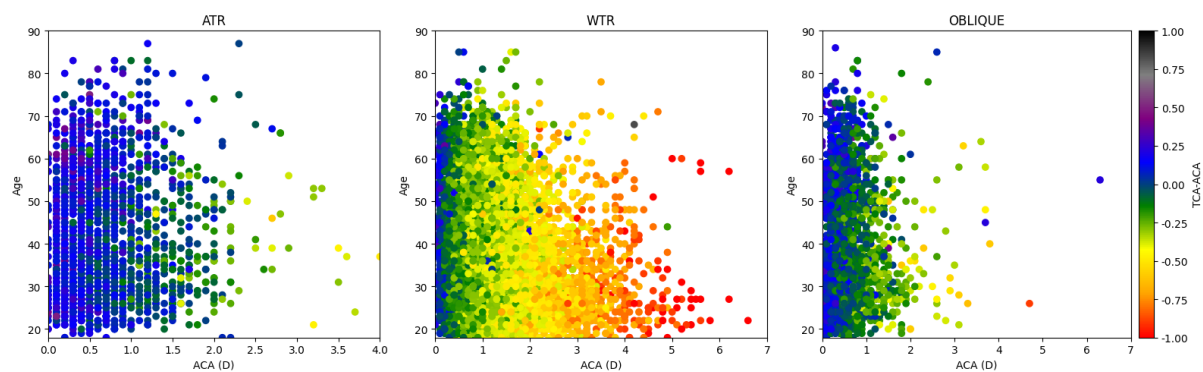

Supplement: Supplementary file 1 [file jcm-13-05617-s001.zip › Supplemental Figure S2.pdf]

**TCA Axis Predictions Scattergram**

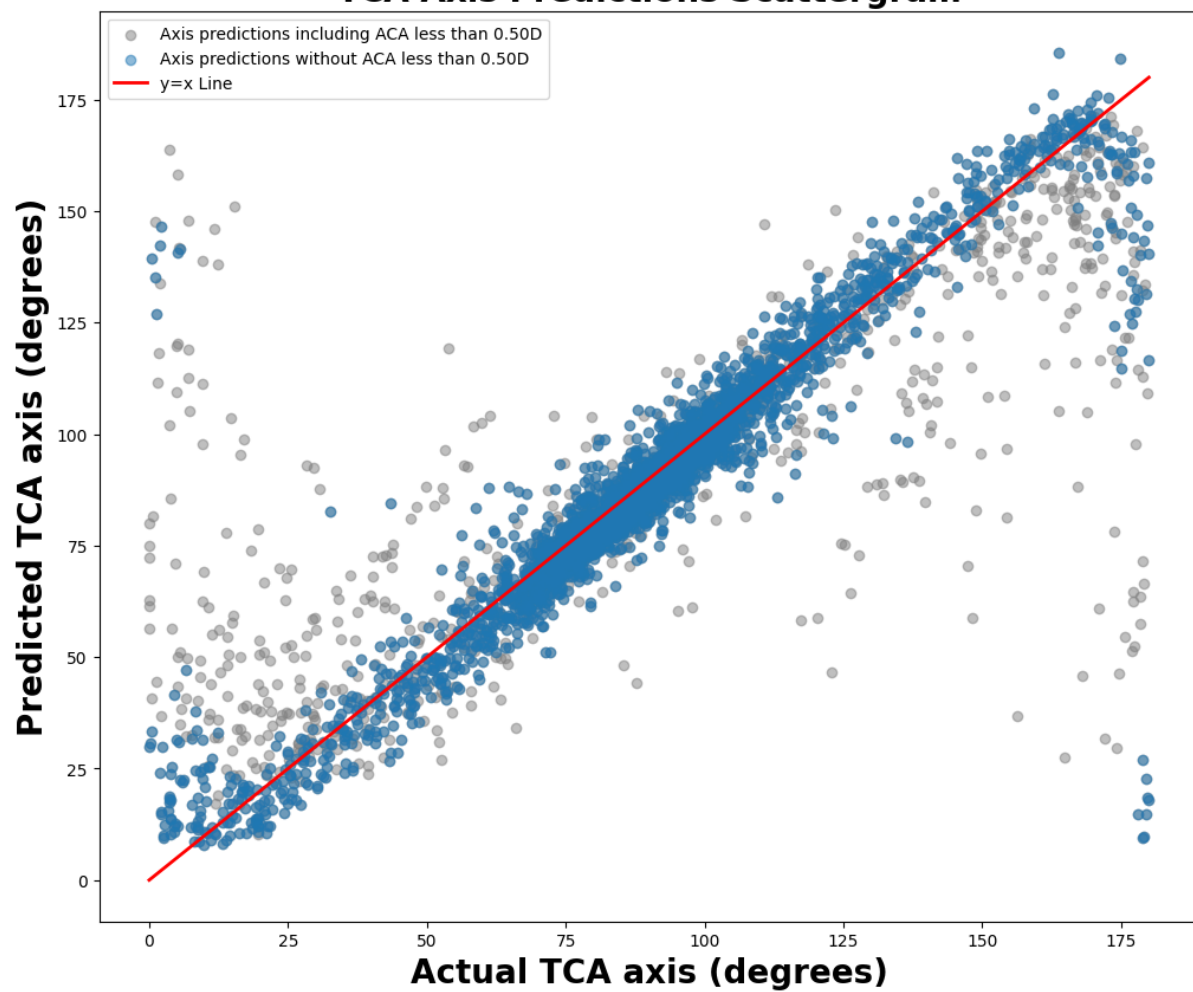

Supplement: Supplementary file 1 [file jcm-13-05617-s001.zip › Supplemental Figure S3.pdf]
